# Longitudinal metabolite profiling of *Streptococcus pneumoniae*-associated community-acquired pneumonia

**DOI:** 10.1007/s11306-024-02091-5

**Published:** 2024-03-05

**Authors:** Ilona den Hartog, Laura B. Zwep, Jacqueline J. Meulman, Thomas Hankemeier, Ewoudt M. W. van de Garde, J. G. Coen van Hasselt

**Affiliations:** 1 Division of Systems Pharmacology & Pharmacy, Leiden Academic Centre for Drug Research, Leiden University, Einsteinweg 55, 2333 CC Leiden, The Netherlands; 2 Metabolomics Centre, Leiden Academic Centre for Drug Research, Leiden University, Leiden, The Netherlands; 3 LUXs Data Science, Leiden, The Netherlands; 4 Department of Statistics, Stanford University, Stanford, CA USA; 5 Division of Pharmacoepidemiology and Clinical Pharmacology, Department of Pharmaceutical Sciences, Utrecht University, Utrecht, The Netherlands; 6 Department of Clinical Pharmacy, St. Antonius Hospital, Nieuwegein, The Netherlands

**Keywords:** Community-acquired pneumonia, Metabolomics, Personalized medicine, High-dimensional data, Longitudinal data

## Abstract

**Introduction:**

Longitudinal biomarkers in patients with community-acquired pneumonia (CAP) may help in monitoring of disease progression and treatment response. The metabolic host response could be a potential source of such biomarkers since it closely associates with the current health status of the patient.

**Objectives:**

In this study we performed longitudinal metabolite profiling in patients with CAP for a comprehensive range of metabolites to identify potential host response biomarkers.

**Methods:**

Previously collected serum samples from CAP patients with confirmed *Streptococcus pneumoniae* infection (n = 25) were used. Samples were collected at multiple time points, up to 30 days after admission. A wide range of metabolites was measured, including amines, acylcarnitines, organic acids, and lipids. The associations between metabolites and C-reactive protein (CRP), procalcitonin, CURB disease severity score at admission, and total length of stay were evaluated.

**Results:**

Distinct longitudinal profiles of metabolite profiles were identified, including cholesteryl esters, diacyl-phosphatidylethanolamine, diacylglycerols, lysophosphatidylcholines, sphingomyelin, and triglycerides. Positive correlations were found between CRP and phosphatidylcholine (34:1) (cor = 0.63) and negative correlations were found for CRP and nine lysophosphocholines (cor = − 0.57 to − 0.74). The CURB disease severity score was negatively associated with six metabolites, including acylcarnitines (tau = − 0.64 to − 0.58). Negative correlations were found between the length of stay and six triglycerides (TGs), especially TGs (60:3) and (58:2) (cor = − 0.63 and − 0.61).

**Conclusion:**

The identified metabolites may provide insight into biological mechanisms underlying disease severity and may be of interest for exploration as potential treatment response monitoring biomarker.

**Supplementary Information:**

The online version contains supplementary material available at 10.1007/s11306-024-02091-5.

## Introduction

Community-acquired pneumonia (CAP) is a lower respiratory tract infection with a high incidence and is associated with the hospitalization of approximately one million adults per year (Battleman et al., 2002). The most common cause of CAP is *Streptococcus pneumoniae* (Wiersinga et al., 2018). In hospitalized CAP patients, there is a need to monitor the antibiotic treatment response to optimize the treatment strategy (Pletz et al., 2022). In addition, there is a need for guidance on decisions about earlier termination of antibiotic treatment to minimize the risk of antimicrobial resistance. Monitoring of treatment response is currently achieved through observation of clinical symptoms and with inflammatory markers such as C-reactive protein (CPR) and procalcitonin (PCT) (Aulin et al., 2021; Karakioulaki & Stolz, 2019). In particular, PCT is relevant for informing early treatment termination decisions but lacks predictive performance for CAP prognosis (de Jong et al., 2016; Guo et al., 2018). Therefore, there is a need for biomarkers that give early insights into the clinical course of CAP.

Biomarkers that reflect the current physiological state of the patient have the potential to accurately monitor and predict the treatment response in CAP patients. Because the metabolome closely represents this physiological state, metabolomics-techniques may enable discovery of relevant novel biomarkers. Indeed, for CAP and sepsis, the potential for metabolomics-based biomarkers measured at a static time point has been demonstrated (Seymour et al., 2013). However, the longitudinal monitoring of metabolic changes within patients may allow for an improved characterization of treatment response (Kohler et al., 2017). For example, CAP patients show a change in lysophosphatidylcholines that mirrors the transition from acute illness to recovery after starting antibiotic treatment (Müller et al., 2019). Further systematic characterization of longitudinal metabolic changes in CAP patients may thus be of relevance for identification of metabolic biomarkers that can predict and monitor the treatment response in these patients.

To this end, in this study, we aimed to comprehensively characterize the change of longitudinal metabolite profiles in hospitalized CAP patients with a confirmed *S. pneumoniae* infection using metabolomic profiling and evaluate how metabolic changes relate to disease severity based on CURB scores, established inflammation markers, and clinical treatment response quantified using the length of stay in the hospital.

## Materials and methods

### Patient cohort

We utilized serum samples collected at multiple time points during hospitalization from 25 hospitalized CAP patients with an *S. pneumoniae* infection. These samples were previously collected as part of a larger clinical study that was performed between November 2007 and September 2010 (Meijvis et al., 2011). The causative pathogen was identified using blood or sputum cultures, or a urinary antigen test. We selected samples from patients with a confirmed *S. pneumoniae* infection. We excluded patients with a mixed infection involving additional pathogen(s) and one patient that died during the study period. Samples were collected at five time points: at the day of admission (day 0), and at days 1, 2, 4, and 30 after admission. CRP and creatinine were measured in the hospital at the same time points as the blood samples used for metabolite profiling obtained. Samples were stored at − 80 °C, and went through a maximum of 2 freeze–thaw cycles, so stable metabolites were preserved in the samples (Breier et al., 2014; Goodman et al., 2021). Not all time points were available for each patient, resulting in 115 samples over the 25 patients. On the day of admission, disease severity was determined using the CURB score, which is a scoring system based on confusion, blood urea > 7 mmol/l, respiratory rate (RR) ≥ 30/min; systolic BP < 90 mmHg or diastolic BP ≤ 60 mmHg (Neill et al., 1996). A score of two or higher is classified as severe CAP.

### Bio-analytical procedures

Serum samples were analyzed using five targeted LCMS methods and one targeted GCMS method by the Biomedical Metabolomics Facility of Leiden University, Leiden, The Netherlands, as described previously (Hartog et al., 2021). The metabolite profiling covered 596 metabolite targets from 25 metabolite classes, including amino acids, biogenic amines, acylcarnitines, organic acids, and multiple classes of lipids. Details of the metabolomic analysis methods used are provided in the Supplementary Information. A total of 369 unique metabolites was measured as relative levels, of which 6 metabolites were removed due to high missingness (≥ 20%), resulting in 363 metabolites being evaluated in data analysis. Biochemically-selected sums and ratios of metabolites were calculated and added to the data (Supplementary Table 1).

PCT was measured in the same serum samples used for the metabolite profiling analysis. PCT analysis was performed using the human procalcitonin CLIA kit from Abbexa (abx190129). Samples were measured in duplicate if sample volumes were sufficient (95% of samples).

### Data analysis

The metabolite levels were scaled through log-transformation and standardization. To explore the variability of the high-dimensional metabolite profiling dataset, principal component analysis (PCA) was used. The PCA was used on the scaled metabolite profiling data over the different time points, with the metabolites as variables and each observation being a sample from a patient for a specific time point (Van Der Ham et al., 1997). As part of the PCA, missing values were imputed through multiple imputation using expectation maximization (EM-PCA), which iteratively calculates the principal components and imputes the missing values (Josse et al., 2011).

To evaluate how much of the variation in the metabolites could be explained by the change over time, the first two principal components were related to time using a polynomial regression model. The importance of the metabolites to explain the variation between the patients over time was evaluated by evaluating the squared variable loadings. Specifically, the squared variable loadings within and between biochemical metabolite classes were evaluated to study similarities within classes and see which biochemical classes vary more between the patients.

To characterize the metabolic time profiles and profiles of current inflammation markers for different patients, we estimated the correlations between the scaled metabolite levels and CRP, PCT and creatinine levels over time. Next, we evaluated which metabolites could be of interest for the prediction of the clinical course, by estimating the Kendall’s Tau correlation between the scaled metabolite levels and a clinical disease severity marker, the CURB score (Neill et al., 1996) at hospital admission, and estimating Pearson correlation between the scaled metabolite levels the outcome length of stay (LOS) in the hospital. Since the CURB and LOS are static values, while the metabolites changed over time, the correlations between these outcomes and the change in metabolite levels from baseline (m_t=k_ − m_t=0_) at each time point (k) were calculated. Due to the large number of correlations calculated and the small sample size, the correlations were not tested for significance, to prevent multiple testing problems, instead we choose an exploratory analysis where the metabolites with the largest (positive or negative) correlation were further evaluated in literature research to assess their biological function.

All analyses were performed in R. The scripts used for the analyses were deposited on GitHub (http://github.com/vanhasseltlab/LongitudinalMetabolomicsCAP/tree/manuscript).

## Results

### Metabolite time profiles

Metabolite profiling was performed for 25 patients and resulted in 363 metabolite levels on five time points (Supplementary Table 2). The patient characteristics are displayed in Table 1. Comorbidities present in patients included kidney disease (n = 1), cardiovascular disease (n = 4), malignancy (n = 2), COPD (n = 1, n_missing_ = 15), diabetes (n = 3, n_missing_ = 15). No patients were using corticosteroids before admission (n_missing_ = 15).

**Table 1:** Patient characteristics

	CAP patients (N = 25)
Age (years)	
Median [min, max]	67.0 [18.0, 98.0]
Sex	
Male	12 (48.0%)
Female	13 (52.0%)
CURB score	
Median [min, max]	1.00 [0, 3.00]
Duration of symptoms before admission (days)	
Median [min, max]	3.00 [1.00, 14.0]
Missing	15 (60.0%)
Antibiotic treatment before admission	
No	8 (32.0%)
Yes	2 (8.0%)
Missing	15 (60.0%)
Length of stay (days)	
Median [min, max]	7.50 [2.50, 24.5]

**Fig. 1:**
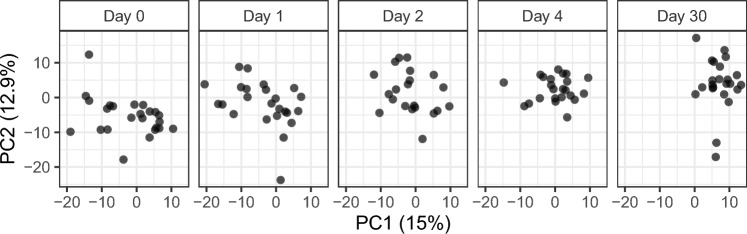
PCA scores for patient metabolite profiles over time. Every point represents the scores of an individual patient at a certain time point, in two dimensions based on the metabolite values. The panels show a trend over time of the metabolite profiles

Metabolite profiles within all CAP patients shifted over time, as shown in the PCA over all time points (Fig. 1). The close relationship between metabolite levels and time is reflected in the results from the polynomial regression model which indicated that 45% of the metabolite variation captured in these first two principal components could be explained by time. Due to the large age range, we tested whether age was a large explanatory factor for the metabolite differences between individuals, but did not find a significant contribution of age (Supplementary Information).

**Fig. 2:**
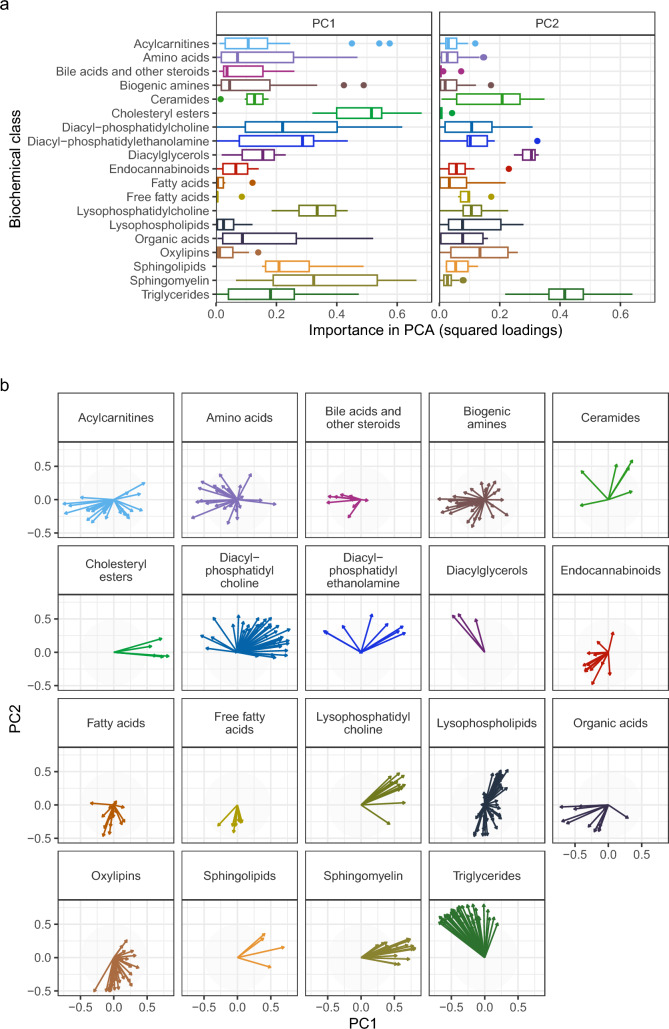
Metabolite contributions to the two dimensions of the PCA as variable loadings. **A** The importance of each biochemical class for the different principal components (PCs), expressed by their squared metabolite loadings. Each box represents the squared loadings of the metabolites within a metabolic class. High squared loadings indicate a larger contribution to explaining the variation between patients. **B** The loading plots for each biochemical metabolite class. The arrows indicate the importance (length) and direction of the metabolites in the principal component space. For example, high PC1 values correspond to high metabolite levels for metabolites with right pointing arrows, and low metabolite levels for metabolites with left pointing arrows. Arrows with a similar direction have similar metabolite patterns. *PC* principal component

The metabolites that were targeted in this study were categorized into different biochemical classes. Metabolites from different biochemical classes showed distinct contributions to the total variation between the patients over time as was expressed in the variable loadings and directionality of the principal components (Fig. 2). The squared PCA loadings represent the weight that the different metabolites in the biochemical class have in explaining the variation between patients over time. Of the variation in principal component one and two, 48% was explained by metabolites of the classes of cholesteryl esters, LPC’s, sphingomyelins, diacylglycerols, and triglycerides (Fig. 2A). The metabolites were categorized in classes based on their biochemistry and not based on their biological functions. The PCA indicate that metabolites that are categorized in the same class do not necessarily behave similarly (Fig. 2B). For example, amino acids behave very differently from each other. Metabolites that do behave similarly in their biochemical class are for example triglycerides and sphingomyelins.

**Fig. 3:**
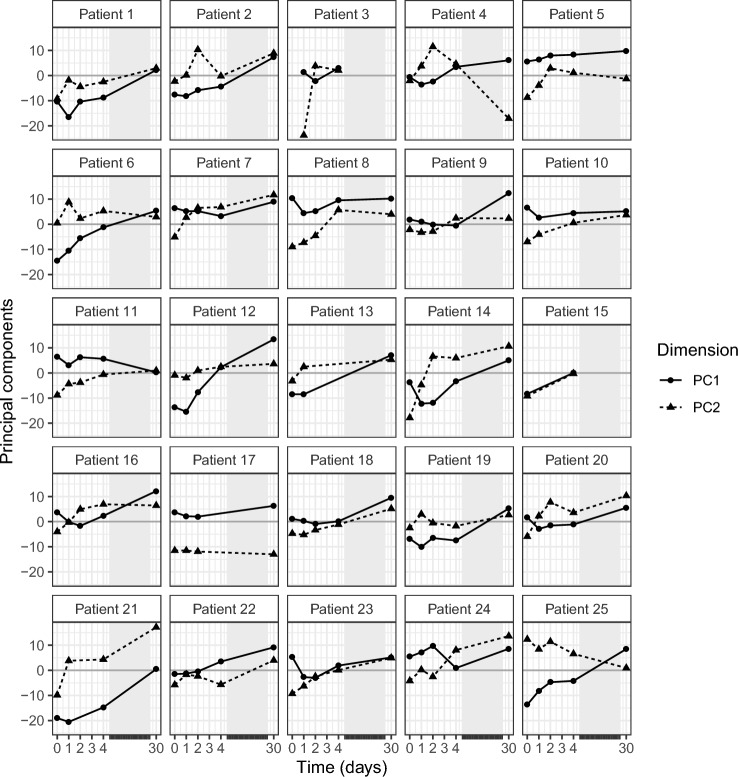
Individual time profiles over PC1 and PC2. The lines PC1 (solid) and PC2 (dashed), indicate the change in the corresponding principal component over time. Changes in PC values correspond to changes in metabolite levels according to their respective loadings. *PC* principal component

For each patient, the metabolic time profiles were shown as the two first components from the PCA (Fig. 3; Supplementary Fig. 1). Generally, a shift from low to high principal component values was seen over time, corresponding to the shift in metabolite levels for the different metabolites (Fig. 2B). The large variability in the time profiles indicates a large interpatient variability in metabolic levels and changes over time.

### Inflammation marker associations

**Fig. 4:**
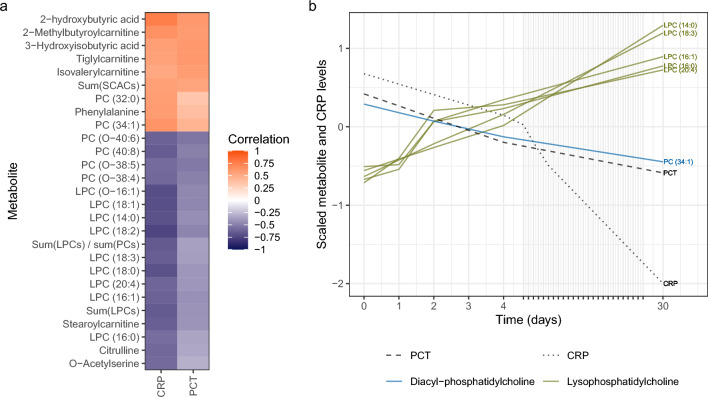
Correlations between inflammation markers CRP and PCT, and metabolites. **A** The correlations between metabolites and CRP or PCT. Metabolites with a correlation > 0.55 or < − 0.55 for at least one marker are shown. A positive correlation (orange) indicates that a higher CRP or PCT level corresponds to an increase of that metabolite over time, while a negative correlation (blue) indicates a decrease over time for patients with a higher CRP or PCT level. **B** Average CRP, PCT, PC (34:1), and LPC levels over time over all patients. Metabolite and CRP data were scaled. Abbreviations: see the abbreviation list

To explore associations between metabolite profiles and inflammation, the metabolite values were compared to currently used inflammation biomarkers. Correlations were found between CRP and PCT and several metabolites. For example, phosphocholine (PC) (34:1) showed a positive correlation with CRP (cor = 0.63). Several individual lysophosphocholines (LPCs) and the sum of all LPCs showed a negative correlation with CRP (cor = − 0.57 to − 0.74, Fig. 4A). PC (34:1) was found to decrease over time and several LPCs showed an increase over time, thereby mirroring the clinical disease progression (Fig. 4B). Positive correlations with CRP and PCT were reported for the short-chain acylcarnitines (SCACs) tiglylcarnitine, 2-methylbutyroylcarnitine, and isovalerylcarnitine (cor with PCT = 0.61, 0.58, and 0.57; cor with CRP = 0.54, 0.64, and 0.51, respectively). Negative correlations were seen between the long-chain acylcarnitine (LCAC) stearoylcarnitine and CRP (cor = 0.62). This trend for decreasing SCACs over time is also represented by the positive correlation of CRP and PCT with the sum of all SCACs (cor = 0.55 and 0.53, respectively).

Correlations between metabolite levels and creatinine, a marker of renal failure, were also identified. The same trends were seen for creatinine as for CRP and PCT (Supplementary Fig. 2). Strong positive correlations were observed between creatine and 1-Methylhistidine, SDMA, inositol, homoserine, methionine sulfone, and octanoylcarnitine (cor > 0.7).

### Disease severity score associations

**Fig. 5:**
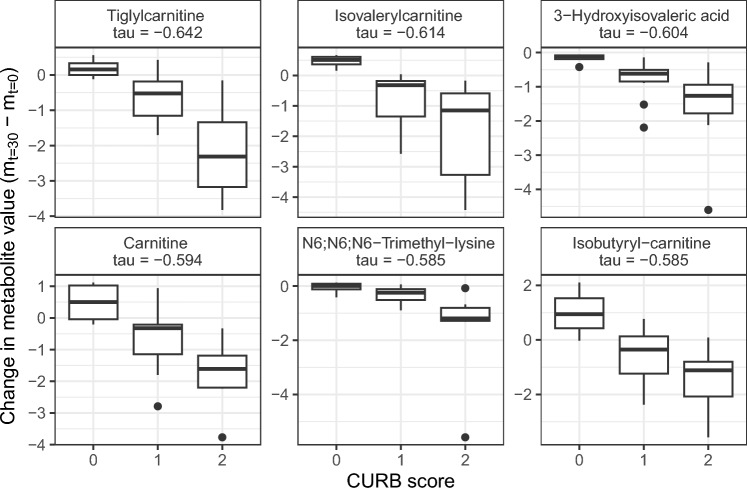
The correlation between the CURB score and six metabolites with highest associations**.** The change in metabolite level is the difference between the scaled metabolite level at day 30 and scaled metabolite level at admission (y-axis). These six metabolites all show a negative correlation with the CURB score (tau). This means, for patients with a CURB score of 0 the metabolite change between day 30 and day 0 is positive, so their metabolite levels were increasing over time. For patients with a CURB score of 2, the metabolite levels decreased over time

To identify possible metabolic biomarkers for indication of disease severity, associations between the CURB disease severity score at admission and the change in metabolite levels on from day 0 to days 1, 2, 4, and 30 were evaluated (Supplementary Fig. 2). Negative associations were found between the CURB score and the change of metabolite levels (m) between day 0 and day 30 (m_t=30_ − m_t=0_) of tiglylcarnitine, isovalerylcarnitine, 3-hydroxyisovaleric acid, carnitine, *N*6,*N*6,*N*6-trimethyl-lysine, and isobutyryl carnitine (tau = − 0.64 to − 0.58, Fig. 5). Patients with higher CURB scores showed decreasing levels of these metabolites.

### Hospital length of stay associations

**Fig. 6:**
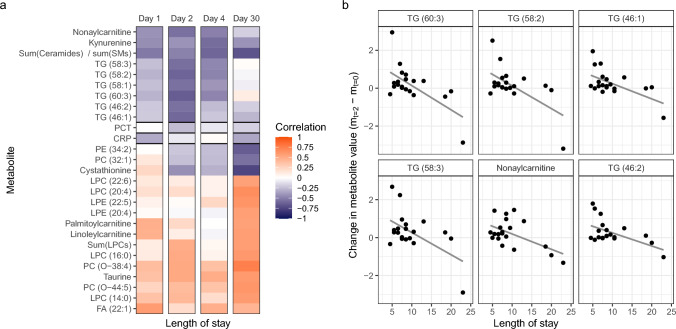
**A** The correlations between the length of stay and metabolite change from baseline at days 1, 2, 4, and 30 after admission (m_t=k_ − m_t=0_). CRP and PCT are added as a reference. A positive correlation (orange) indicates that a longer stay in the hospital corresponds to an increase of that metabolite over time, while a negative correlation (blue) indicates a decrease over time for patients with longer stay. **B** Metabolite levels over time for individual patients for metabolites with large negative correlations (cor < − 0.55) over the first 2 days after admission. Abbreviations: see the abbreviation list

We evaluated the association between metabolites and clinical outcomes using the length of stay (LOS) as a potential surrogate endpoint. The strongest negative correlations to LOS were reported for the metabolite change over the first two days of admission (m_t=2_ − m_t=0_, Fig. 6), especially for the triglycerides (TGs) (60:3) and (58:2) (cor = − 0.63 and − 0.61 respectively). The correlations of these metabolites to LOS were much stronger than to CRP and PCT (cor = − 0.08 and − 0.25 respectively). Positive correlations were most pronounced when analyzing the metabolite change from the day of admission to day 30 (m_t=30_ − m_t=0_). In the case of fatty acid (FA) (22:1) the day after admission (m_t=1_ − m_t=0_) was the most strongly positively correlated to the LOS (cor = 0.58).

## Discussion

In this study, we characterized the dynamics of the serum metabolites in pneumococcal CAP patients. We found that a large part of the variation in the metabolite values was associated with time-varying changes in metabolites within the patients. We furthermore found that several groups of metabolites were found to correlate with inflammation markers, CURB score, and length of hospital stay. These findings both support the potential relevance of metabolite-based biomarkers to monitor the treatment response or disease progression in CAP.

We found that length of stay in the hospital was negatively correlated with the triglycerides TG (60:3) and TG (58:2). Interestingly, these TGs are not highly correlated to CRP, PCT, or the CURB score, which suggests that they can explain a part of the variability of disease progression in patients not explained by established biomarkers for inflammation. We previously found that TGs do not contribute to the etiological prediction of pathogenic in CAP (Hartog et al., 2021); as such TGs may be of interest as potential biomarker beyond pneumococcal CAP studied in this analysis. Further studies should however consider the potential impact of diet on TGs, as a potential confounding factor (Parks, 2001).

Phosphatidylcholine (PC) (34:1) and lysophosphatidylcholines (LPCs) (14:0), (16:0), (16:1), (18:0), (18:1), (18:2), (18:3) and (20:4) correlated to inflammatory markers, which also corresponds to previous findings (Banoei et al., 2020; Müller et al., 2019). PC (34:1), a ligand of nuclear receptor PPARα30, showed a positive correlation with CRP, which was previously associated with an anti-inflammatory response (Colombo et al., 2018). LPC 14:0 has been recently identified as a biomarker for disease severity in CAP patients (Nan et al., 2022). Due the correlation with CRP, these metabolites could be of interest as treatment response biomarkers, also beyond pneumococcal CAP patients (Saleh et al., 2019).

The CURB score was negatively associated with six metabolites, including several acylcarnitines. One of these acylcarnitines, tiglylcarnitine, has previously been found to be increased in non-survivors of CAP and could be considered a marker for disease severity (Banoei et al., 2020). Isovalerylcarnitine and isobutyrylcarnitine have, to our knowledge, not been studied as disease severity marker before, but may show a comparable performance to tiglylcarnitine as their direction on the first principal component is similar.

In this analysis we demonstrated which biochemical metabolite classes explain most of the variation in metabolite patterns between individuals and over time. Triglycerides and LPCs were important for explaining the variation over time in the principal component analysis (PCA) and correlated with LOS and inflammatory markers. Within the biochemical classes, not all metabolites showed similar patterns, indicating that metabolites in some biochemical classes behave similarly during the infection, while metabolites in other classes behave differently (Fig. 2B). The amino acids behave very differently, which could be expected since they are involved in a wide variety of biological functions (Wu, 2009).

The longitudinal analysis of the metabolite profiling data enabled us to gain insight into acute and longer-term changes in the metabolome during the clinical course of CAP. Since patients are admitted to the hospital in different stages of the disease, interpretation of the metabolite profile at one time point can be challenging. The longitudinal metabolite profiles that were measured in this study give more information about the state of the patient and elucidate the effect of comorbidities and co-medications. The principle component analysis (Fig. 3) showed large variability between different patients, indicating the importance of considering changes within patients, instead of evaluating the metabolite profile at one timepoint. We found that the differences in metabolite levels were largely explained by changes over time and were, therefore, related to the treatment response.

The clinical samples used in this study were stored approximately 10 years before metabolomics measurements were performed, with a maximum of 2 freeze/thaw cycles. The prolonged storage duration of these samples may have affected the metabolite levels present in these samples. Our study design does not allow to assess the extent of this impact. Prior studies suggest on the effect of storage age on plasma metabolite levels within 7 years of storage was found negligible (Wagner-Golbs et al., 2019). Moreover, despite any potential storage effects, we expect that relative ratios of unique metabolites within and between samples will remain constant.

This study was conducted in a well-characterized set of CAP patients with *S. pneumoniae* infections. *S. pneumoniae* is a common cause of CAP, but other bacterial or viral pathogens can also be the cause of CAP. A previous study did not show significant differences in metabolic profiles between common causes of CAP (den Hartog et al., 2021). The results of the current study may apply to CAP patients with these other causative pathogens, but this is still unsure because the previous study does not cover changes over time. Especially metabolites associated with length of stay should be validated in CAP cohorts with various causative pathogens, since they are not related to the general inflammatory response.

In further research, the addition of patients with other causes of CAP is of interest to compare metabolic time profiles for different treatment strategies based on the causative pathogen. Early recognition of a pathogen-drug mismatch using metabolite profiling could make antibiotic therapies more targeted and shorter. This study shows that mainly TGs, LPCs, PCs, and acylcarnitines are of interest for the disease severity and the length of stay for patients with CAP. By focusing on these metabolite classes, the number of metabolites that has to be measured for every patient can be reduced.

In conclusion, we find that that metabolomics-based biomarkers have potential for treatment response monitoring in CAP patients. The triglycerides found in this study could potentially complement the currently available biomarkers such as CRP and PCT as they yield additional information about the clinical course in these patients. This study furthermore supports the relevance for collecting longitudinal data to follow the highly dynamic metabolite profiles in patients, which can further enable the development of personalized treatment strategies.

### Supplementary Information

Below is the link to the electronic supplementary material.

Supplementary file1 (PDF 11 kb)

Supplementary file2 (PDF 15 kb)

Supplementary file3 (CSV 2 kb)

Supplementary file4 (CSV 10 kb)

Supplementary file5 (PDF 147 kb)

## Data Availability

The data in this study were anonimized and are made available on GitHub at https://github.com/vanhasseltlab/LongitudinalMetabolomicsCAP/tree/manuscript as an R data object.

## Abbreviations

BP: Blood pressure
BUN: Blood urea nitrogen
CAP: Community-acquired pneumonia
COPD: Chronic obstructive pulmonary disease
cor: Correlation
CRP: C-reactive protein
EM-PCA: Expectation maximization-principal component analysis
FA: Fatty acids
LCAC: Long-chain acylcarnitine
LOS: Length of stay
LPC: Lysophosphatidylcholine
LPE: Lysophosphatidylethanolamine
PC: Phosphatidylcholine
PCA: Principal component analysis
PCT: Procalcitonin
PE: Phosphatidylethanolamine
RR: Respiratory rate
SCAC: Short-chain acylcarnitine
SDMA: Symmetric dimethylarginine
SM: Sphingomyelin
TG: Triglycerides
