# Supplementary material for: Longitudinal metabolite profiling of Streptococcus pneumoniae-associated community-acquired pneumonia
Source: Metabolomics. 2024 Mar 5;20(2):35. doi: 10.1007/s11306-024-02091-5 (PMC10914916; doi:10.1007/s11306-024-02091-5)
Supplement: Supplementary file 1 — Supplementary file1 (PDF 11 kb) [file 11306_2024_2091_MOESM1_ESM.pdf]

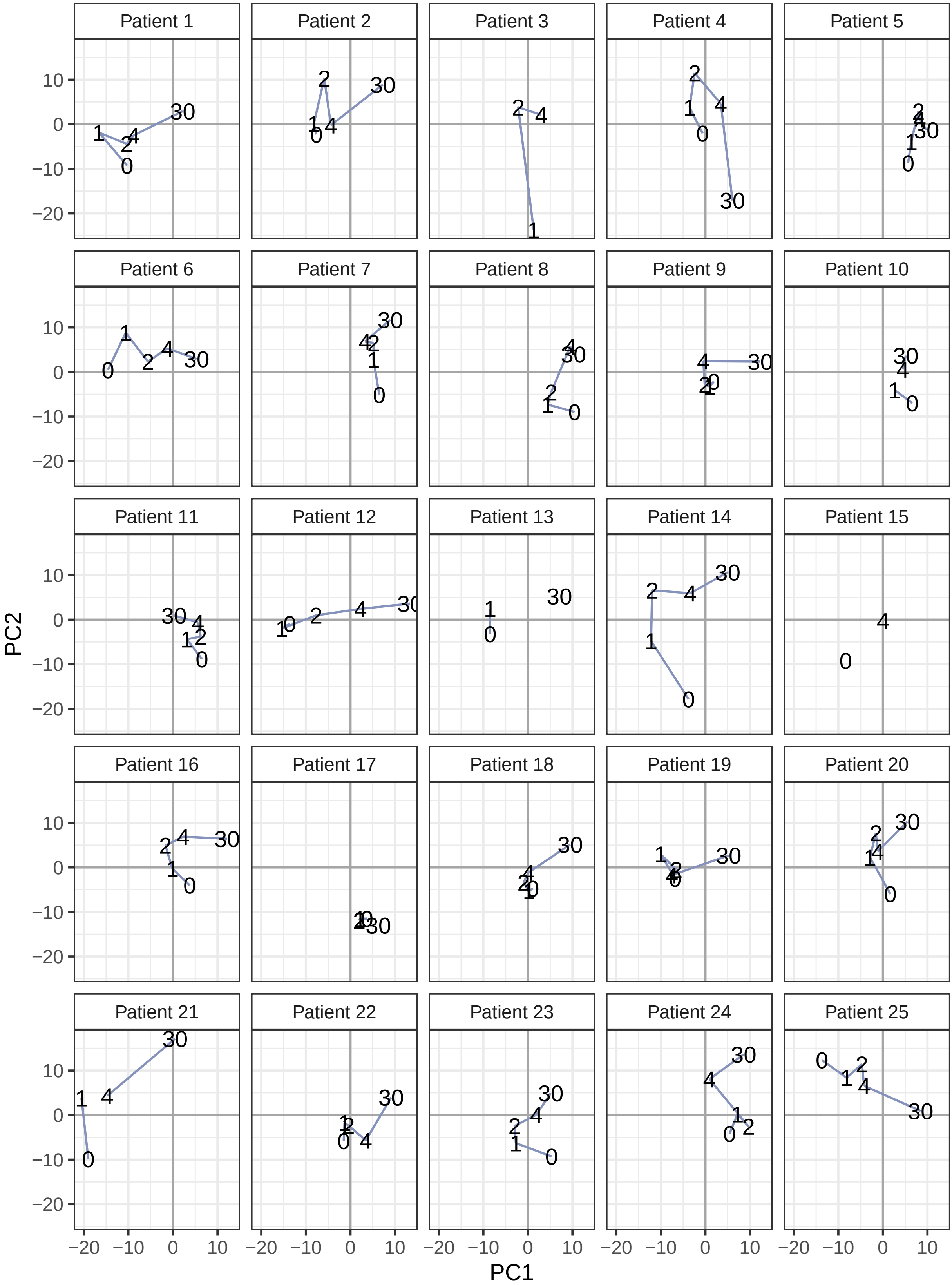

Figure S1. PCA score plots for each patient. For each patient, the time points are labelled and connected with lines. Abbreviations: PC: principal component
