# Supplementary material for: Longitudinal metabolite profiling of Streptococcus pneumoniae-associated community-acquired pneumonia
Source: Metabolomics. 2024 Mar 5;20(2):35. doi: 10.1007/s11306-024-02091-5 (PMC10914916; doi:10.1007/s11306-024-02091-5)
Supplement: Supplementary file 2 — Supplementary file2 (PDF 15 kb) [file 11306_2024_2091_MOESM2_ESM.pdf]

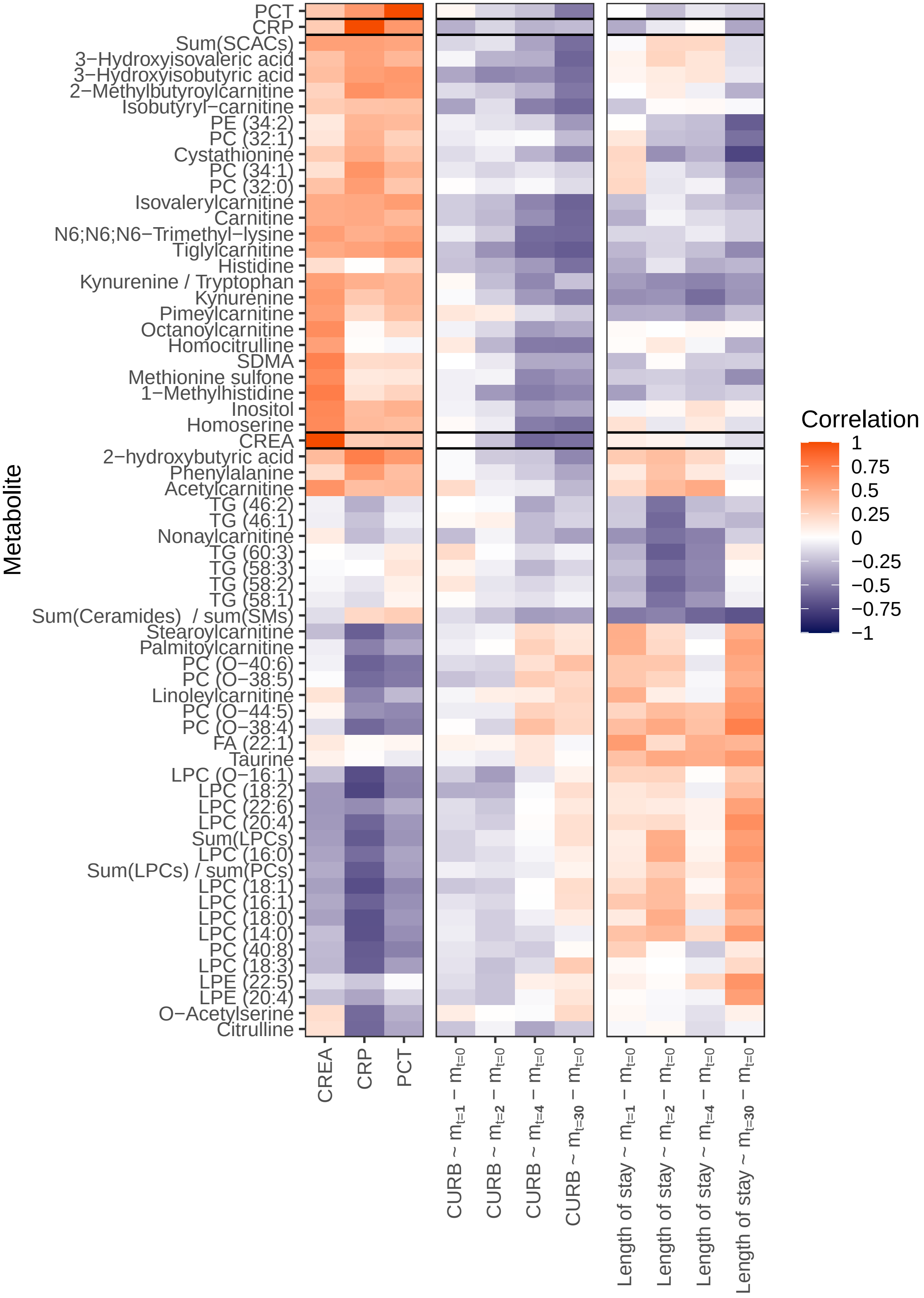

Figure S2. The correlations between metabolites and creatinine, CRP, and PCT over time; and the correlations of the CURB score and length of stay with a change of the metabolites between day k and day 0, where the change in metabolite levels is denoted by mt=k   mt=0.
